# MOBILITY TRAJECTORIES, HEALTHCARE SATISFACTION, AND PERCEIVED DISABILITY DISCRIMINATION AMONG OLDER ADULTS

**DOI:** 10.1093/geroni/igz038.1928

**Published:** 2019-11-08

**Authors:** Collin Mueller, Jessica S West

**Affiliations:** Duke University, Durham, North Carolina, United States

## Abstract

Although functional mobility limitations are associated with increased healthcare needs in later life, little research explores how older adults with varying functional mobility trajectories experience healthcare quality. To this end, we explore the effects of functional mobility trajectories on differences in healthcare treatment satisfaction, perceived disability discrimination in healthcare settings, and perceived everyday disability discrimination. We analyzed 9 waves of the Health and Retirement Study (n=29,284, 1998-2014, ages 50-84). First, we estimate age-specific group-based trajectories of functional mobility across age using finite mixture models. Second, we use multinomial logistic regression to identify sociodemographic factors that place individuals at elevated risk of membership in each group. Third, we explore how membership in one disability trajectory group over another affects healthcare satisfaction, perceptions of everyday discrimination in the context of healthcare treatment settings, and perceived discrimination in everyday life. Regression models include clustered standard errors to account for heteroscedasticity across repeated observations of individuals over time. We identify six group-based trajectories of functional mobility limitation among aging Americans. Black, female, and less-educated Americans are at higher risk of membership in disadvantaged trajectories, characterized by more rapidly increasing counts of functional mobility limitations, than their counterparts. Disadvantaged functional limitation trajectories are associated with lower levels of healthcare satisfaction, higher levels of perceived physical disability discrimination in healthcare treatment settings, and higher levels of perceived physical discrimination in other contexts of everyday life. The present study advances our knowledge of how older adults experience healthcare settings and discrimination across functional mobility status trajectories.

